# Changes in Physicochemical Properties and Qualities of Red Brown Rice at Different Storage Temperatures

**DOI:** 10.3390/foods10112658

**Published:** 2021-11-02

**Authors:** Tao Wang, Nana She, Mengnan Wang, Bo Zhang, Jiaxing Qin, Jingyuan Dong, Guozhen Fang, Shuo Wang

**Affiliations:** 1 State Key Laboratory of Food Nutrition and Safety, Tianjin University of Science and Technology, Tianjin 300457, China; ahzytaowang@163.com (T.W.); S13652006697@163.com (N.S.); wanmn0000@163.com (M.W.); zhangbo0083@126.com (B.Z.); qinjiaxing1995@163.com (J.Q.); 16622880324@163.com (J.D.); s.wang@tust.edu.cn (S.W.); 2 Tianjin Key Laboratory of Food Science and Health, School of Medicine, Nankai University, Tianjin 300071, China

**Keywords:** red brown rice, storage temperature, physicochemical property, quality

## Abstract

The effects of storage temperature on the physicochemical properties and qualities of red brown rice were investigated in this study. The samples were vacuum-packed in nylon/polyethylene pouches and stored at 15 °C, 25 °C and 35 °C for 12 weeks. The moisture content decreased as storage time was prolonged. Rice stored at 15 °C and 25 °C had a lower falling range of water content compared to the samples stored at 35 °C. Free fatty acid values increased fastest when samples were stored at a high temperature, and the rise can be effectively delayed at low temperatures. The pH of residual cooking water and adhesiveness decreased, while the heating water absorption rate and hardness increased during storage for red and brown rice. Low-field nuclear magnetic resonance results indicate that water molecules migrated, the binding force of H protons became stronger and the bonds between molecules became closer with increased storage duration. Temperature had an obvious correlation with starch granules and protein structure, characterized by a scanning electron microscope and Fourier transform infrared spectroscopy. Low temperatures significantly retarded those changes. The results indicate that storage temperature is a vital factor affecting the physicochemical properties and qualities of red brown rice and provided reference and theoretical basis for the actual storage of red brown rice.

## 1. Introduction

Rice (*Oryza sativa* L.) is a staple food for more than 60% of the world’s population, especially in Asia [1]. As a major dietary source of carbohydrates, rice plays a critical role in meeting energy needs and nutrient intake [2]. At present, with the progress of society and the development of the economy, people’s living standards are improving day by day, and awareness of nutrition and health is constantly being enhanced. The concept of food consumption has also undergone a huge change. People’s consumption of rice is no longer a simple solution to food and clothing, but a transformation of nutrition, healthcare and food therapy. Colored rice has gradually come into public view due to its rich nutrition and good medicinal effects. Red rice is one of the most widely studied types of colored rice. It is not only rich in nutrients, but also has the functions of activating blood circulation, eliminating stasis and moisturizing skin, and it has anti-aging properties as well as benefits in healthcare, etc. [3]. Since ancient times, it has been considered a good nourishing product and a good homologous substance of medicine and food, which is favored by consumers and has great market potential [4].

Brown rice is the whole fruit of unshelled rice, which is composed of four parts: the cortex, aleurone layer, embryo and endosperm. Brown rice is rich in nutrients and natural biological active substances (such as γ-aminobutyric acid, γ-glutamyl alcohol, glutathione, ceramide, etc.) that white rice lacks because it retains the germ and rice bran layer. These are confirmed to have anticancer properties and prevention and curing effects for ailments such as of diabetes, high cholesterol and obesity in human health, and disease prevention and the treatment of modern civilization is of great significance [5]. Therefore, brown rice is listed as a whole-grain healthy food by the U.S. Food and Drug Administration (FDA), which advocates eating it straight. Storage is a necessary stage from harvest to rice consumption and should be carefully controlled because a large number of physical, chemical and physiological changes occur during storage, which greatly affect the final quality of rice [6,7]. However, due to the loss of the protection given by the rice husk, brown rice embryos and endosperms are exposed and easily damaged by mechanical mechanisms. The increase in enzyme activity led to the acceleration of lipid degradation during storage and the decline of brown rice quality [8,9,10]. At the same time, brown rice is susceptible to insect mildew, which reduces its edible and economic value.

At present, the research on red rice mainly focuses on red rice’s epidermal pigment [11], biological activity [12], red rice products and other effects. The planting areas and yield of red rice are increasing continuously. However, there have been no literature reports on suitable storage conditions for red rice. Hence, it is vital to select the suitable packaging materials and packaging methods for storing red brown rice. However, the traditional plastic woven bags and other packaging materials are not adequate barriers or moisture-proof, thus increasing the possibility of red brown rice mildew during storage. Generally, rice qualities were mainly evaluated by the changes of starch gelatinization and texture [13]. Meanwhile, the changes of water content, microstructure and protein secondary structure can directly reflect the quality of rice [14,15]. In this study, red brown rice was vacuum-packed in nylon/polyethylene little pouches, which can adequately protect the red brown rice from the outside environment and ensure the quality of red brown rice. Therefore, the change in physical and chemical indices and the quality of red brown rice under the condition of vacuum packaging was studied in this paper. It aims to provide technical reference and theoretical basis for the actual storage of red brown rice and the findings can be popularized and applied to the storage of colored rice, effectively solving the problem of grain storage, achieving safe grain storage and providing an effective storage method both for farmers who plant colored rice and for consumers, so as to delay the deterioration of its quality and reduce losses.

## 2. Materials and Methods

### 2.1. Chemicals and Reagents

The red brown rice was purchased from Jinggangshan in the Jiangxi province and harvested in October 2019. The vacuum bags were purchased from Tmall stores and were made of nylon/polyethylene composite. The specifications and the water vapor transmission were 12 × 20 cm and 0.5 g/(m^2^/24 h), respectively. N-hexane was purchased from Aladdin Biochemical Technology Co., LTD (Shanghai, China). Other chemicals were purchased by Sinopharm Chemical Reagent Co., LTD (Tianjin, China). N-hexane was of chromatographic grade. The grade of the other regents and chemicals was analytical and used with no additional purification.

### 2.2. Red Brown Rice Storage

Temperature and humidity were the most vital environmental factors influencing rice quality during storage. Typically, high-humidity storage conditions would easily cause rice mildew [16,17]. To satisfy consumer demand, harvested red brown rice must be stored for extended periods under conditions that maintain its quality. In this strategy, red brown rice was repacked into vacuum bags weighing 200 g each and placed in an artificial climate box for simulated storage. A low room-temperature and high temperature were set at (15, 25 and 35 °C), respectively, for each sample, and the humidity was set at 65%. The storage period was 12 weeks. Samples were taken every 2 weeks and a series of indices were measured.

### 2.3. Determination of Moisture Content and Fatty Acid Value of Red Brown Rice

Moisture content was determined by drying the red brown rice flour to a constant weight at 105 °C (American Association of Cereal Chemists, 2000), and fatty acid value was determined using the official standard method (American Association of Cereal Chemists, 1976).

### 2.4. LF-NMR Experiments

The red brown rice sample was weighed at about 3 g, then wrapped in plastic wrap and put into a 25 mm diameter sample tube. The height of the sample in the tube should not exceed 2 cm. The reference material of soybean oil was used to calibrate the instruments. A nuclear magnetic resonance analyzer (MicroMR-025, Newmai Electronic Technology Co., LTD, Suzhou, China) was used to acquire the transverse relaxation time (T_2_) of the sample. The waiting time (TW), the echo time (TE) and the number of echoes (NECH) were 1500 ms, 0.1 ms and 4000, respectively.

### 2.5. Determination of Cooking Characteristics of Red Brown Rice

#### 2.5.1. Determination of Heating Water Absorption Rate of Red Brown Rice

A total of 10 g (*w*_0_) of red brown rice was accurately weighed and placed in an aluminum box. Distilled water that was twice the weight of the red brown rice was added, and the rice was soaked for 0.5 h. We removed and drained the remaining water on the surface of the rice grains with filter paper, then added distilled water that was 1.4 times the weight of the red brown rice, covered the aluminum box and put it on the steamer of a boiled pot, heated it for 30 min, kept it warm for 15 min, took it out and weighed the rice (*w*_1_).

$$\mathrm{heating}\;\mathrm{water}\;\mathrm{absorption}\;\mathrm{rate}\left(\%\right)=\frac{w_{1}-w_{0}}{w_{0}}\times 100$$

#### 2.5.2. Determination of pH Value of Red Brown Rice Leach

We weighed the 3 g of red brown rice cooked in Section 2.5.1 and put it into a colorimetric test tube of 25 mL, added water to a volume 12.5 mL, oscillated it in a water bath at 40 °C for 1 h, then added 25 mL distilled water at a constant volume, shook it well and centrifuged it at 3000 r/min for 10 min. After centrifugation, 5 mL of supernatant was sucked out and put into a 5 mL beaker. The pH value of the red rice leach was measured with a pH meter.

### 2.6. The Texture Properties of Cooked Red Brown Rice

A texture analyzer (Stable micro systems Co., Godalming, UK) which was loaded with 10 g of trigger force was used to measure the texture characteristics of cooked red brown rice. The probe of the texture analyzer was an aluminum cylindrical probe (P/36 R). The pre-test speed, test speed and post-test speed were 4.0 mm/s, 1.0 mm/s and 1.0 mm/s, respectively. The degree of deformation was set to 30% of the sample height. Four full grains of red rice were selected from the cooked rice and placed on the platform of the texture apparatus. The experiment was repeated at least three times in parallel, and the average value was taken to analyze the results.

### 2.7. Scanning Electron Microscopy

Scanning electron microscopy (SEM) was used to observe the changes in the cross-section microstructure of red brown rice during storage. Red brown rice with a relatively smooth cross section and natural fractures during each storage period was selected as the sample to be tested. The sample was fixed on the vertical sample paste board with conductive adhesive, and the cross section of the rice sample was taken upward. Finally, the sample was characterized by a scanning electron microscope at a magnification of 1000×.

### 2.8. Determination of Secondary Structure of Proteins

The relative content of protein secondary structures was obtained by a Fourier transform infrared spectrometer (IS50, Thermo Nicolet Co., LLC, Waltham, MA, USA). Red brown rice was milled and sifted through 120 mesh. An accurately weighted sample of 1.0 mg was added into a mortar containing 150 mg KBr. Then, the mixture was grinded into pellets for analysis. The spectrograms were collected with a wave number range of 4000–400 cm^−1^ [18]. The spectra in the region of 1700–1600 cm^−1^ were deconvolved by OMINC 8 (Thermo, Waltham, MA, USA).

## 3. Results and Discussion

### 3.1. Moisture Content

**Figure 1:**
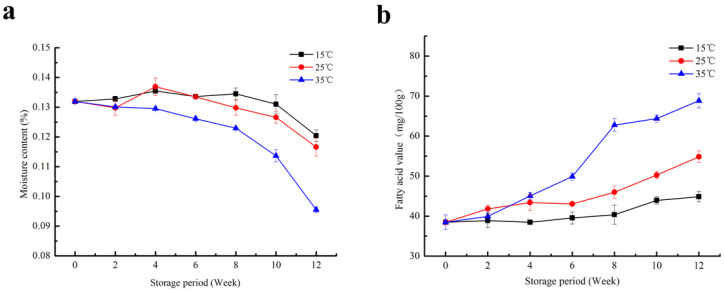
Changes in physicochemical indices of red brown rice under different storage temperature ((**a**): moisture content; (**b**): fatty acid values).

Moisture content is one of the most important indicators in measuring the freshness and quality of red rice. It can not only determine whether red rice can be stored safely, but also affects the retention of the aroma of red rice, and it is closely related to the final edible quality [19]. An appropriate water level can closely combine with starch, protein and other hydrophilic colloids so as to improve the edible quality. Rice with a high moisture content can induce the survival and growth of pests and microorganisms. Mold and microorganisms generally increase in proportion with the moisture content [20]. Too low a moisture content will cause the grain to break and reduce its viscosity after cooking, which will affect its taste and edible quality. As shown in Figure 1a, the moisture content of red brown rice stored at 15 °C and 25 °C did not change significantly in the first 8 weeks, and even showed a slight increase. The main reason for the increase in moisture content was due to the moisture exchange between the rice and its surroundings. It showed a gradually decreasing trend from the eighth week. After 12 weeks, the moisture contents decreased to 12.04% and 11.67%, respectively. The moisture content showed a continuously decreasing trend, and finally decreased to 9.57% when stored at 35 °C for 12 weeks. High temperatures can enhance the enzyme activities and respiratory metabolism in the grains of red brown rice, enhance the water exchange between the outside and the brown rice, accelerate the rate of water loss, and thus accelerate the deterioration of the quality of brown rice. Therefore, it is significant and necessary to monitor the changes in the water content of red brown rice at different storage temperatures during the storage period.

### 3.2. Fatty Acid Value

The changes in the free fatty acid values of the red brown rice with storage time are shown in Figure 1b. During storage, the fatty acid values increased gradually, and it is obvious that the fatty acid values of the red brown rice stored at a high temperature (35 °C) were higher than those of rice stored at lower temperatures (15 °C and 25 °C) (Figure 1b). Although a gradual increase in fatty acid values (from 38.48 mg/100 g to 44.86 mg/100 g) was observed in red brown rice stored at 15 °C, much higher values were obtained in red brown rice stored at 25 °C (54.86 mg/100 g) and 35 °C (68.83 mg/100 g) after 12 weeks of storage. Lipid content is one of the most important components of rice. The deterioration degree of rice quality during storage can be reflected by the change in fatty acid value, which is one of the important indices to measure the freshness and aging degree of rice. Too much accumulation of fatty acids during storage will lead to rice rancidity and seriously degrade its edible quality [21]. The influence of temperature on fatty acid value is mainly due to the influence of temperature on enzyme activity. Within a certain range, the higher the temperature, the stronger the enzyme activity, which leads to a faster rate of fat decomposition and a faster increase in fatty acid value.

### 3.3. The LF-NMR Experiments

**Figure 2:**
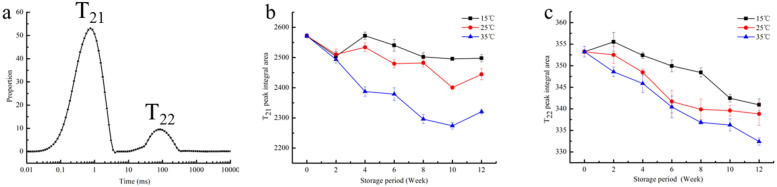
Distribution of T_2_ relaxation time of fresh red brown rice (**a**) and changes in T_21_ (**b**) and T_22_ (**c**) peak area in red brown rice at different temperatures.

Low-field nuclear magnetic resonance (LF-NMR) has become an alternative and effective method to quantify the changes in water status and visualize the internal water distribution of food [22,23]. In order to further study the moisture distribution and moisture relaxation behavior of red brown rice during storage, this study used the Carr–Purcell–Meiboom–Gill(CPMG) pulse sequencing to measure the transverse relaxation time (T_2_). The value of T_2_ can reflect the motion of H protons, and different values of T_2_ represent different degrees of freedom of the water [24]. The T_2_ inversion map is used to distinguish the moisture with different fluidity in the form of peaks. Different peaks represent different fluidity of water molecules. When the binding force of water molecules is weak, the corresponding T_2_ value is large, and the peak in the inversion map is to the right [25,26]. From Figure 2a, we can see two peaks, indicating that there are two kinds of water molecules with different fluidity in the fresh red brown rice. The peak range of the first peak is 0.1–10 ms, and the peak area proportion is more than 85%, which is called a T_21_ peak. The peak range of the second peak is 10–1000 ms, which is called a T_22_ peak. The T_21_ and T_22_ peaks represent bound and free water in red brown rice, respectively. The area of peak T_21_ is significantly larger than that of peak T_22_; that is, the bound water content is significantly higher than the free water content. This is because the free water is mainly lost by the drying of brown rice before storage, and the bound water is not easy to lose due to the close combination. As can be seen from Figure 2b,c, with the extension of storage time, the peak areas of T_21_ and T_22_ both show a decreasing trend, indicating that water content gradually decreases in the storage process, and the decreasing rate is related to temperature. The higher the temperature, the higher the rate of decline, which was in accord with the above conclusion of moisture content determination. The peak area of T_21_ decreased from 2571.78 to 2479.74 when stored at 15 °C. At 25 °C and 35 °C, the peak area decreased to 2444.37 and 2320.28, respectively. A high temperature will accelerate the vibration frequency of the H proton, which will lead to the easy loss of water. Moreover, red rice will be heated and cause pests, which will increase the amount of broken rice and deteriorate its quality. Therefore, the storage temperature should be strictly controlled in the storage process.

As can be seen from Table 1, with the extension of storage time, the peak point time of T_21_ and T_22_ decreased. In other words, the peak shifted to the left, and the degree of shift varied with the temperature. This indicated that during the storage process, water molecules migrated, the binding force of H protons became stronger and stronger, and the bonds between molecules became closer.

### 3.4. Microstructure Characterization

**Figure 3:**
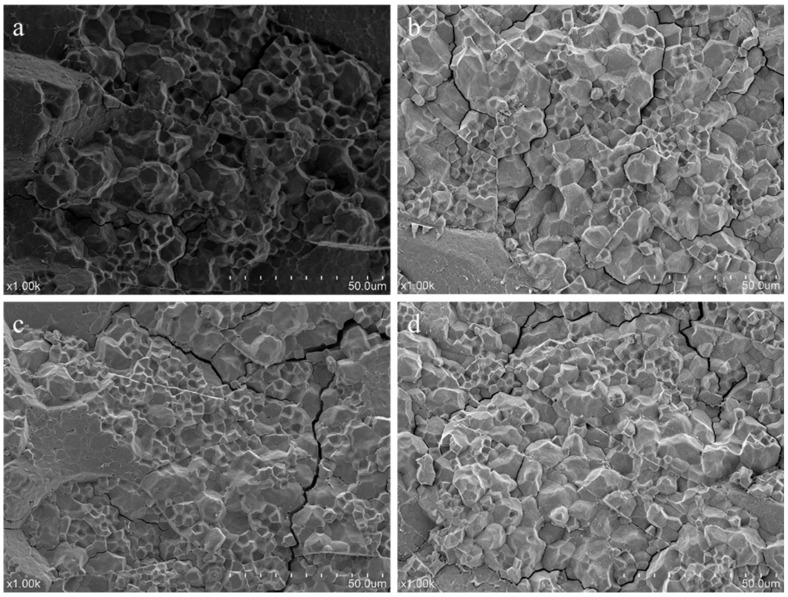
Effect of temperature on the cross-sectional microstructure of red brown rice ((**a**): fresh rice, (**b**): stored at 15 °C, (**c**): stored at 25 °C, (**d**): stored at 35 °C).

The cross-sectional microstructure of fresh red brown rice and red brown rice stored at different temperatures for 12 weeks are shown in Figure 3. Figure 3a shows that the cross section of fresh red brown rice was not completely smooth. Protein, lipid and starch granules were closely linked together. Protein was embedded in starch granules, and these mostly existed in the form of complex granules, with only a few single starch granules. However, regardless of the complex or single granules, the edges and corners of starch granules were clearly visible and showed polyhedral crystal structures that were arranged closely and in an orderly fashion. The boundaries between starch granules were clear and distinct. According to Figure 3b–d, the cross section of brown rice stored at 15 °C and 25 °C did not change significantly. It still existed in the form of complex granules and was arranged neatly, which was close to the initial state as a whole. However, when stored at 35 °C, a few cracks and small holes appeared in the cross section, and some starch granules became blurred. At the same time, the number of single granules increased. The crack may be caused by the decomposition of starch by the debranching enzyme. During the storage process, different degrees of water loss occurred in the rice grains, resulting in the accumulation of starch particles and an increase in density. Eventually, gaps appeared around the granules and the edges of the granules became blurred.

### 3.5. FTIR Analysis

In the infrared spectrum analysis, the peaks at 1700–1600 cm^−1^ represent the amide I band of protein. It is known as being the most valuable for studying the secondary structure of proteins because it includes four different kinds of secondary structures: α-helices, β-sheets, β-turns and randomly coiled structures, which are connected by the C•O stretching of peptide bonds under various environment [27,28].

The changes in protein secondary structure during the storage of red brown rice under different storage conditions were observed more accurately by FTIR, and the FTIR data were analyzed by Peak Fit 4.12. The results are shown in Table 2. From the table, it can be seen that the proportion of β-turn in fresh red brown rice was the highest, followed by β-sheet, and finally by α-helix and random coil, indicating that β-turn was the main protein secondary structure in red rice. In the storage process, with the increase in time, the relative content of the α-helix decreased gradually, random coil increased gradually, and the relative content of β-turn and β-sheet fluctuated without specific laws and trends but still occupied large proportions, which were the main existing forms. The stability of the α-helix structure is closely related to non-covalent bonds, such as hydrogen bonds, which can be influenced by temperature, pH and other factors. The decrease in α-helix structure content was caused by hydrogen bond fracturing. The increase in random coil content indicated that brown rice protein developed from an ordered state to disordered state during storage, and its stability decreased.

### 3.6. Determination of Cooking and Texture Characteristics

**Figure 4:**
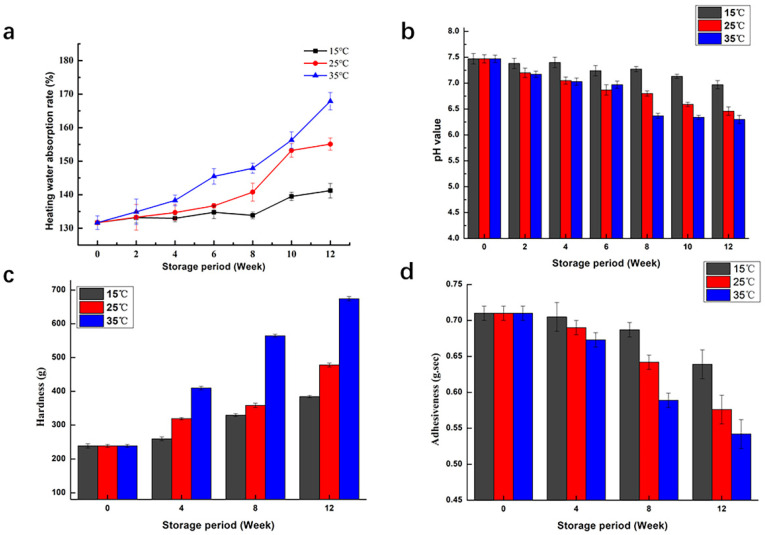
Effect of temperature on cooking and texture characteristics of red brown rice ((**a**): heating water absorption rate, (**b**): pH value, (**c**): hardness, (**d**): adhesiveness).

To investigate the impacts of storage temperature on the cooking characteristics of red brown rice, the heating water absorption rate and pH of residual cooking water were evaluated. Figure 4a,b shows that during the storage of red brown rice, the heating water absorption rate increased, and the pH of residual cooking water decreased over the storage time. Additionally, temperature had a noteworthy effect on cooking characteristics. The higher the temperature was, the higher the rate of change. High temperatures will accelerate the aging rate. Due to the hardening of the cell walls during the aging process of rice, more water was needed to maintain the polygonal crystal shape during the starch gelatinization process, so the water absorption increased gradually with the extension of storage time. During storage, lipase or microorganisms decomposed non-starch lipids to produce a large number of free fatty acids that accumulated in the rice grains, and the decomposition speed was accelerated under high-temperature conditions. In the process of cooking, acid was dissolved in water, which increased the acidity of the residual cooking water and decreased the pH value.

Hardness and adhesiveness served as important indices for evaluating the texture of the cooked rice [29]. The influence of temperature and storage duration on the hardness and adhesiveness are shown in Figure 4c,d. Data confirmed that the hardness of red brown rice increased and its adhesiveness decreased after storage, which was consistent with the conclusion drawn by Zhou [30]. Compared with low and room temperature storage, the change rate of texture properties under high temperature conditions was the fastest and most obvious. In the storage process, amylopectin was decomposed, the crystal structure of starch became more compact, and starch granule strength increased, which might be the reason for the increase in the hardness of cooked rice after storage [31]. In addition, the leaching of soluble starch was inhibited by the formation of complex fatty acids with amylose and protein or the polymerization of amylose molecules themselves, which reduced the rice adhesiveness after long-term storage [32]. Low temperatures can effectively delay the deterioration of red brown rice.

## 4. Conclusions

Changes in the physicochemical characteristic of red brown rice, such as moisture content and distribution, fatty acid value, microstructure and cooking and texture characteristics, apparently occurred during storage under different temperatures. Storage temperature was an important factor affecting the ageing process. With the prolongation of storage time, red brown rice experienced different degrees of aging, which was accelerated at high temperatures and effectively delayed at low temperatures. The selection of appropriate storage conditions can maximize the quality of red brown rice and reduce the waste caused by improper storage. Storage at low temperatures or short-term storage at room temperature is advised to maintain the quality of red brown rice. The present study is helpful in providing farmers and consumers with a storage basis for red rice, so as to maintain grain quality and reduce resource waste caused by improper storage.

## Figures and Tables

**Table 1:** Changes in T_2_ peak time of red brown rice at different temperatures.

Storage Time (Week)	T_21_ (ms)			T_22_ (ms)		
	15 °C	25 °C	35 °C	15 °C	25 °C	35 °C
0	0.76	0.76	0.76	84.26	84.26	84.26
2	0.76	0.66	0.76	79.42	72.36	70.69
4	0.76	0.66	0.66	76.14	69.08	69.08
6	0.66	0.66	0.63	66.94	65.73	69.08
8	0.66	0.66	0.57	66.22	63.37	60.88
10	0.66	0.66	0.57	60.08	62.94	57.22
12	0.66	0.63	0.50	60.08	57.86	49.73

**Table 2:** The effect of temperature on the secondary structure of red brown rice protein.

Storage Temperature	Storage Time (Week)	α-Helix	β-Sheet	β-Turn	Random Coil
	0	0.14	0.30	0.41	0.15
15 °C	4	0.10	0.28	0.46	0.16
	8	0.13	0.27	0.45	0.15
	12	0.11	0.33	0.35	0.21
25 °C	4	0.13	0.28	0.40	0.19
	8	0.12	0.31	0.42	0.15
	12	0.09	0.34	0.39	0.18
35 °C	4	0.13	0.29	0.40	0.18
	8	0.10	0.25	0.42	0.21
	12	0.08	0.32	0.40	0.20

## Data Availability

Not applicable.
